# Serum levels of vitamin D in people with albinism from Brazil^☆^

**DOI:** 10.1016/j.abd.2024.10.003

**Published:** 2025-03-17

**Authors:** Carolina Reato Marçon, Lilian Lemos Costa, Maria Paula Ribeiro Mazzon, Nathalia Terumi Kawakami, Camila Cardoso Paes Carvalho

**Affiliations:** Dermatology Service of Santa Casa de São Paulo, São Paulo, SP, Brazil

*Dear Editor,*

Oculocutaneous albinism is an autosomal recessive disorder caused by mutations in the TYR, AOC2, TYRP1, and SLC45A2 genes, leading to reduced or absent melanin production in melanocytes.^1^ Melanin absorbs and scatters ultraviolet radiation (UVR) and visible light in the skin.^2^ Due to the lack of melanin, individuals with albinism are highly susceptible to the harmful effects of UVR, making sun protection a priority to prevent actinic damage and malignant skin neoplasms.^1^, ^3^, ^4^ Photoprotection reduces vitamin D (VD) production by blocking its synthesis in the skin.^3^, ^5^ Since people with albinism strictly avoid the sun, they are thought to be at risk for low VD levels.^6^, ^7^

Vitamin D is a fat-soluble vitamin mainly obtained from endogenous skin production and, to a lesser extent, from the diet. Most serum VD comes from the conversion of 7-dehydrocholesterol into vitamin D3 in the skin through UVB radiation.^8^ Besides its role in bone metabolism, VD participates in the functions of almost all body systems.^9^ Deficiency has been linked to various diseases, making it important to detect and treat VD deficiency in high-risk groups.^10^

The primary objective of this study was to evaluate serum VD levels in people with albinism who were advised on strict photoprotection and without oral VD supplementation. The secondary objective was to determine the impact of other variables on serum VD levels. This is a prospective cross-sectional observational study conducted at the Dermatology Sector of Santa Casa de São Paulo. Participants were randomly recruited from the Pró-Albino Program. Inclusion criteria included having albinism and no VD supplementation for at least six months. Exclusion criteria included pregnancy and certain comorbidities.

Blood collections were carried out from September 2020 to August 2022. São Paulo has high solar radiation throughout the year, with an average UVB index of 11. Collections were conducted without considering the four climatic seasons. Participants signed informed consent forms, and the project was authorized by the Research Ethics Committee of the Faculty of Medical Sciences of Santa Casa de São Paulo. Participants were interviewed using a sun exposure questionnaire. Daily sun exposure, sunscreen use, mechanical photoprotection measures, and occupation were evaluated. Skin photoaging was assessed using the GLOGAU scale, adapted for albinism, and dietary VD intake was evaluated using a food frequency questionnaire. Demographic and clinical data were collected during medical consultations.

Data considered included age, sex, socioeconomic level, place of birth, skin color, hair color, eye color, smoking, Body Mass Index (BMI), and physical activity. Five milliliters of venous blood were collected for VD analysis. Serum 25(OH)D levels were measured using the ARCHITECT-OH-Vitamin-D chemiluminescence microparticle immunoassay. Data were analyzed using Jamovi® in an *R* environment.

Continuous data were summarized by mean values, confidence intervals, and standard deviation. Categorical data were described by their absolute frequency and proportion. Continuous data were tested for normality using the Shapiro-Wilk test, and parametric or non-parametric tests were applied accordingly.

The sample consisted of 42 individuals with an average age of 22.03 years, average weight of 61.8 kg, average height of 1.51 m, and average BMI of 25 (Table 1). Gender distribution was similar, with 52.4% female. The most common skin color was white (76.3%), and the most common parental skin color was brown (53.4%). Most participants reported using sunscreen (85.7%) and practicing photoprotective measures. The average serum 25(OH)D level was 30 ng/mL.

**Table 1:** Results of constitutional variables.

	N	95% Confidence interval			Standard deviation	Minimum	Maximum
		Mean	Lower limit	Upper limit			
Age	42	27.74	22.03	33.44	18.306	3	67
Weight	42	61.75	53.03	70.47	27.982	13.20	115.00
Height	42	1.51.	1.44	1.58	0.232	1.00	1.83
BMI	42	24.97	22.81	27.13	6.921	12.69	35.13

Details of the constitutional variables (age, weight, height and BMI) found in the 42 individuals in the sample.

Only one participant had VD levels below 20 ng/mL, with 97.6% having levels above 20 ng/mL and 40% above 30 ng/mL (Fig. 1). No significant correlations were found between serum VD levels and variables such as age, sex, skin color, physical activity, or photodamage degree (Table 2). Vitamin D levels were similar between seasons. The data were subjected to a one-way ANOVA test, which confirmed that there was no statistically significant difference in vitamin D levels between the seasons, p = 0.687 (Fig. 2).

**Fig. 1:**
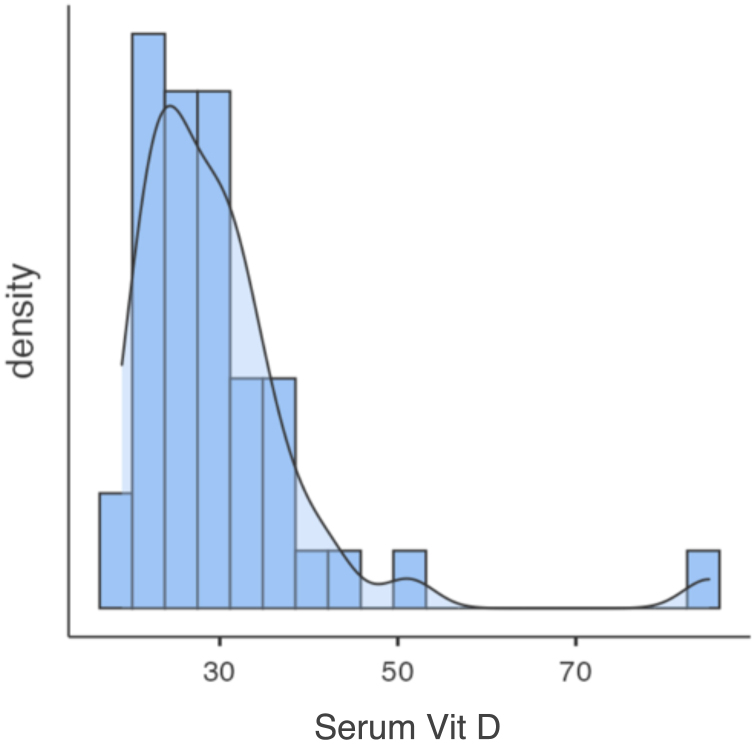
Distribution of vitamin D levels in the sample. The average serum level of 25(OH)D in the sample was 30 ng/mL, with 97.6% of participants having levels above 20 ng/mL, 60% 25(OH)D levels below 30 ng/mL, and 40% above 30 ng/mL, with a maximum value of 85 ng/mL.

**Table 2:** Variables studied and their relationship with serum 25(OH)D levels.

Predictor	Estimates	Standard error	*t*	p
Intercepto	24.43451	7.17522	3.40540	0.002
Vitamin D total	−1.66e-4	0.00277	−0.05997	0.953
White hair color				
White hair ‒ others	0.02573	4.02660	0.00639	0.995
Green eye color				
Green eyes ‒ others	3.02581	4.96927	0.60890	0.547
Daily sun exposure from 6 am to 6 pm ‒ more than 30 min				
More than 30 min ‒ less than 30 min.	0.00130	5.78696	2.24e-4	1.000
Use of sunscreen lotion				
Yes ‒ No	2.75324	6.34766	0.43374	0.667
Sun damage level (choice = type 1 [level])				
Non verified – Verified	2.15289	3.97541	0.54155	0.592

None of the variables tested in this model interfered with the serum levels of 25(OH)D.

**Fig. 2:**
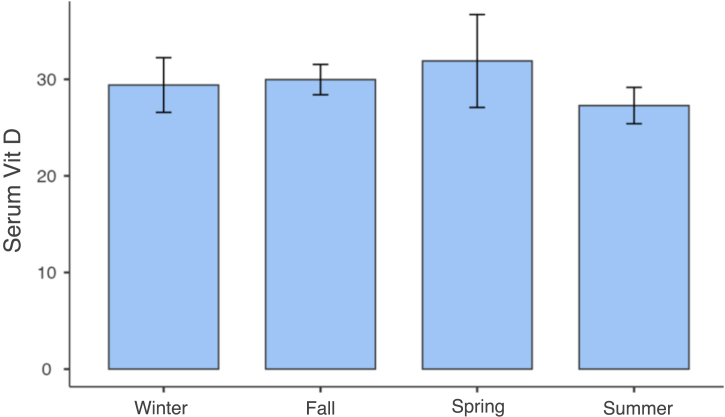
Serum vitamin D levels in different climatic seasons. Vitamin D levels were similar between seasons. The data were subjected to a one-way ANOVA test, which confirmed that there was no statistically significant difference in vitamin D levels between the seasons (p = 0.687).

Only three studies published in the literature specifically evaluated serum vitamin D levels in people with albinism, all of which were carried out in African countries. Namely, these three studies concluded that serum levels of vitamin D in people with albinism, when compared to those with pigmented skin, were higher, even with the supposed photoprotection.^4^, ^5^, ^7^ Similar findings were observed in this Brazilian study, with most participants having normal VD levels despite photoprotective measures. The lack of correlation between VD levels and analyzed variables might be due to the small sample size. More extensive studies with larger samples and detailed analysis could provide further insights.

Serum VD levels in the studied population with albinism were within the sufficiency range, even without oral supplementation and despite photoprotective measures. These values were not influenced by the analyzed variables. People with albinism in regions with high solar radiation are likely not at risk for VD deficiency, and normal VD values should be considered equivalent to the general population. Empirical supplementation is not indicated unless based on individual needs assessed through clinical investigation and periodic measurements.

## Financial support

None declared.

## Authors’ contributions

Carolina Reato Marçon: Approval of the final version of the manuscript; Critical literature review; Data collection, analysis and interpretation; Effective participation in research orientation; Manuscript critical review; Preparation and writing of the manuscript; Statistical analysis; Study conception and planning.

Lilian Lemos Costa: Approval of the final version of the manuscript; Critical literature review; Data collection; analysis and interpretation; Preparation and writing of the manuscript; Study conception and planning.

Maria Paula Ribeiro Mazzon: Approval of the final version of the manuscript; Data collection, analysis and interpretation; Study conception and planning.

Nathalia Terumi Kawakami: Approval of the final version of the manuscript; Data collection, analysis and interpretation; Study conception and planning.

Camila Cardoso Paes Carvalho: Approval of the final version of the manuscript; Data collection, analysis and interpretation; Study conception and planning.

## Conflicts of interest

None declared.
